# Low copy numbers of complement C4 and homozygous deficiency of C4A may predispose to severe disease and earlier disease onset in patients with systemic lupus erythematosus

**DOI:** 10.1177/0961203317735187

**Published:** 2017-10-19

**Authors:** M Jüptner, F Flachsbart, A Caliebe, W Lieb, S Schreiber, R Zeuner, A Franke, J O Schröder

**Affiliations:** 1 First Clinic of Internal Medicine, University Hospital of Kiel, Kiel, Germany; 2 Institute of Clinical Molecular Biology, Christian-Albrechts-University of Kiel, Kiel, Germany; 3 Institute of Medical Informatics and Statistics, Christian-Albrechts-University of Kiel, Kiel, Germany; 4 Institute of Epidemiology, Christian-Albrechts-University of Kiel, Kiel, Germany

**Keywords:** Systemic lupus erythematosus, complement C4, copy number variation, HLA

## Abstract

**Objectives:**

Low copy numbers and deletion of complement *C4* genes are potent risk factors for systemic lupus erythematosus (SLE). However, it is not known whether this genetic association affects the clinical outcome. We investigated *C4* copy number variation and its relationship to clinical and serological features in a Northern European lupus cohort.

**Methods:**

We genotyped the *C4* gene locus using polymerase chain reaction (PCR)-based TaqMan assays in 169 patients with SLE classified according to the 1997 revised American College of Rheumatology (ACR) criteria and in 520 matched controls. In the patient group the mean C4 serum protein concentrations nephelometrically measured during a 12-month period prior to genetic analysis were compared to *C4* gene copy numbers. Severity of disease was classified according to the intensity of the immunosuppressive regimens applied and compared to *C4* gene copy numbers, too. In addition, we performed a TaqMan based analysis of three lupus-associated single-nucleotide polymorphisms (SNPs) located inside the major histocompatibility complex (MHC) to investigate the independence of complement *C4* in association with SLE.

**Results:**

Homozygous deficiency of the *C4A* isotype was identified as the strongest risk factor for SLE (odds ratio (OR) = 5.329; *p* = 7.7 × 10^−3^) in the case-control comparison. Moreover, two copies of total *C4* were associated with SLE (OR = 3.699; *p* = 6.8 × 10^−3^). C4 serum levels were strongly related to *C4* gene copy numbers in patients, the mean concentration ranging from 0.110 g/l (two copies) to 0.256 g/l (five to six copies; *p* = 4.9 × 10^−6^). Two copies of total *C4* and homozygous deletion of *C4A* were associated with a disease course requiring cyclophosphamide therapy (OR = 4.044; *p* = 0.040 and OR = 5.798; *p* = 0.034, respectively). Homozygous deletion of C4A was associated with earlier onset of SLE (median 24 vs. 34 years; *p* = 0.019) but not significant after correction for multiple testing. SNP analysis revealed a significant association of HLA-DRB1*0301 with SLE (OR = 2.231; *p* = 1.33 × 10^−5^).

**Conclusions:**

Our findings confirm the important role of complement C4 genes in the development of SLE. Beyond the impact on the susceptibility for lupus, C4 copy numbers may be related to earlier onset and a more severe course of the disease. The association of homozygous deletion of C4A and SLE is accompanied by the presence of HLA-DRB1*0301 without a proven pathophysiological mechanism.

## Introduction

Systemic lupus erythematosus (SLE) is a complex multi-organ disease of unknown origin causing acute and chronic organ damage. A recent search for genetic factors revealed more than 30 associated genes and loci, almost all of which are attributable to functions of the immune system.^1^ As in most genome-wide association studies, the knowledge of the risk genes identified is limited to the association in general, whereas their causal role in the pathogenesis of the disease remains to be clarified.

Among these genes, the complement *C4* gene with its isotypes *C4A* and *C4B* and the special feature of copy number variation has been repeatedly studied in lupus patients. Especially low copy numbers of *C4* and deletion of *C4A* or *C4B* have consistently been reported as potent risk factors for SLE.^2–4^ However, the impact of specific genetic backgrounds on certain clinical variants of the disease or its clinical course is scarcely known. In our study, we investigated the contribution of genetic variations of complement *C4* to the clinical course of the disease.

Since the *C4* gene locus is part of the highly polymorphic major histocompatibility complex (MHC) gene region, the impact of other genetic factors located inside the MHC possibly confounding the association of *C4* copy number variation and SLE has been discussed controversially. To assess the relative weight of *C4* genes and potentially confounding genes within the MHC, we analyzed three single-nucleotide polymorphisms (SNPs) recently reported to have the strongest association with SLE in British and Spanish patient groups.^3^

## Material and methods

### Study participants

A total of 169 patients with the clinical diagnosis of SLE based on the 1997 revised American College of Rheumatology (ACR) criteria,^5^ who presented at the departments of Rheumatology and Nephrology of the University Hospital of Kiel between 1986 and 2013, were enrolled in this study. Inclusion criteria were a regular and structured follow-up and a treatment period of at least 12 months. The patient group consisted of 160 European individuals, five patients from the Middle East, three patients of Eastern Asian origin and one patient from Togo, Africa.

There were 520 age- and sex-matched unrelated controls drawn from the population-based German biobank POPGEN.^6^ The study was carried out according to the Declaration of Helsinki, written informed consent was obtained from all participants and the study has been approved by the local ethics committee. Ethylenediaminetetraacetate (EDTA) and serum blood samples were taken from all patients and controls.

### Deoxyribonucleic acid (DNA) preparation and *C4* genotyping

EDTA blood samples were stored at −80℃ up to genetic analysis. DNA extraction was performed automatically by the Autopure LS system with Gentra Puregene chemistry (Qiagen, Hilden, Germany). Whole genome amplification was performed using the Illustra GenomiPhi V2 DNA Amplification Kit according to the manufacturer’s guidelines (GE Healthcare, Little Chalfont, UK). *C4* copy number genotyping was performed using the common method based on the TaqMan® real-time PCR technology as described elsewhere (assay description: *C4A*: Hs07226349; *C4B*: Hs07226350; Life Technologies Corporation, Foster City, CA, USA).^7^

In addition, we performed a replication analysis of three recently reported lupus-associated SNPs located inside the MHC locus that appear to be related to the *C4* gene locus (assay description: rs2187668: hCV58662585; rs3135391: hCV2455638; rs558702: hCV940258; Life Technologies Corporation, Foster City, CA, USA).^3^ SNP genotyping was performed using the common TaqMan® SNP Genotyping Assays as described elsewhere.^8^

*C4* concentrations from serum blood samples were measured by nephelometry using the BN II system (Siemens Healthcare, Erlangen, Germany).

### Quality control

Copy number results were assessed using the CopyCaller v1.0 Software (Life Technologies Corporation, Foster City, CA, USA) and were checked for quality according to the recommended procedure obtained from the CopyCaller user manual. In brief, quality control consisted of exclusion of all samples with fewer than three replicates, exclusion of all samples with confidence <0.95 and |CN_predicted_ – CN_calculated_| >0.3, exclusion of all samples with *Z* score ≥2.65 and exclusion of all samples with 2.65 >Z score ≥1.75 and |CN_predicted_ –CN_calculated_| >0.3.^9^

After quality control, 160 (total *C4*), 163 (*C4A*) and 164 (*C4B*) usable copy number values remained in the patient group and 460 (total *C4*), 479 (*C4A*) and 473 (*C4B*) usable copy number values in the control group.

SNP data were successfully collected from 168 (rs558702), 169 (rs2187668) and 165 (rs3135391) patients and 512 (rs558702), 514 (rs2187668) and 513 (rs3135391) controls.

### Clinical features

Because a generally accepted scoring system for the severity of SLE is not available, we decided to use the most intensive treatment given to an individual patient as a surrogate parameter for the severity of the respective disease course. Therapeutic classification consisted of six categories, increasing from no therapy as the lowest to cyclophosphamide treatment as the highest category. In detail, treatment regimens were graded into the following categories: (1) no therapy, defined as the complete absence of any lupus-related medication; (2) treatment with hydroxychloroquine without accompanying prednisolone; (3) prednisolone therapy, either as monotherapy or combined with antimalarials and nonsteroidal antirheumatic drugs; (4) application of methotrexate or azathioprine; (5) administration of mycophenolate or cyclosporine; (6) treatment with cyclophosphamide.

### Statistical analyses

Copy number groups to be compared are based on results found by Yang et al.^4^ Here, significant differences between cases and controls were found for *C4* for 2 vs. >2 and ≥5 vs. <5 and for *C4A* for homozygous deficiency (0 vs. >0), homozygous and heterozygous deficiency (≤1 vs. >1) and for ≥3 vs. <3. In the study presented by Yang and colleagues, a frequency of two copies of *C4* was found in 9.3% of the patients and in 1.5% of the controls (odds ratio (OR) 6.5). With a significance level of 0.05, this yields a power of 97% for the sample size of our study for a two-sided χ^2^ test. Even for a considerable lower OR of, for example, 3.0, the power of our study is a reasonable 58% (PS power v3.1.2 software).

Independence of *C4* gene copy numbers between cases and controls as well as independence of *C4* gene copy numbers and therapeutic status in patients (and their related ORs) were tested with Fisher’s exact test. Independence of *C4* gene copy numbers and peripheral protein concentration/age at first diagnosis were tested with the Kruskal-Wallis test. Results were adjusted for multiple testing using Bonferroni’s correction. The Jonckheere-Terpstra test was performed to elucidate a trend in peripheral protein concentration across *C4* gene copy numbers. Multiple logistic regression with backward selection was performed to investigate the joint influence of *C4/C4A* gene copy numbers (with various groupings) and SNP data on the case-control status. Inheritance models of SNP data were tested with the χ^2^ test.

Statistical analysis was performed using SPSS 19 software (IBM).

## Results

### Patients

The patient group consisted of 154 females and 15 males with a mean age of 47.3 ± standard deviation (SD) 13.8 years, ranging from 19 to 78 years. The control group consisted of 471 females and 49 males with a mean age of 49.1 ± 15.5 years, ranging from 19 to 75 years. A total of 163 patients fulfilled at least four of the 1997 revised ACR criteria and six patients were classified as incomplete SLE with typical lupus-related manifestations matching three ACR criteria. Demographic data and clinical characteristics of the patients are shown in Table 1.

**Table 1:** Demographic data and clinical presentation of 169 patients according to the 1997 revised ACR criteria and the most intensive lupus-related treatment ever applied

*(a)*	*Patients* *(*n = *169)*	*Controls* (n = *520)*
Sex	154 females and 15 males	471 females and 49 males
Mean age	47.3 ± 13.8 years	49.1 ± 15.5 years
Age range	19–78 years	19–75 years
Age at diagnosis	36.6 ± 14.1 years	–
Duration of disease	11.1 ± 8.7 years	–
BMI	25.3 ± 5.1	–
Median ACR criteria	5	–
*(b)*	*ACR criteria*	*Number of patients (frequency)*
	Malar rash	83 (49.1%)
	Discoid rash	66 (39.1%)
	Photosensitivity	63 (37.3%)
	Oral ulcerations	42 (24.9%)
	Arthritis	136 (80.5%)
	Serositis	40 (23.7%)
	Renal involvement	37 (21.9%)
	CNS involvement	13 (7.7%)
	Hematologic disorders:	
	Hemolytic anemia	23 (13.6%)
	Leukocytopenia	49 (29.0%)
	Lymphocytopenia	35 (20.7%)
	Thrombocytopenia	22 (13.0%)
	Immunologic disorders:	
	Antibodies against dsDNA	132 (78.1%)
	Antibodies against Sm	14 (8.3%)
	Antinuclear antibodies	169 (100%)
*(c)*	*Grading of therapy*	*Number of patients (frequency)*
	No therapy (Grade 1)	2 (1.2 %)
	Hydroxychloroquine (Grade 2)	10 (5.9%)
	Prednisolone (Grade 3)^a^	27 (16.0%)
	Methotrexate or azathioprine (Grade 4)	24 (14.2%)
	Mycophenolate or cyclosporine (Grade 5)	53 (31.4%)
	Cyclophosphamide (Grade 6)	53 (31.4%)

Characterization of 169 patients according to the ACR criteria and the most potent treatment ever applied. A: If not otherwise denoted, mean ± standard deviations are given.

a The treatments in this table are listed only in case they represent the most intensive modality used in a given patient, i.e. those patients in categories 4 to 6 receiving concomitant prednisolone were not included in category 3.

ACR: American College of Rheumatology; BMI: body mass index; CNS: central nervous system; dsDNA: double-stranded DNA.

### Gene copy numbers and ORs

The frequencies of *C4* gene copies of total *C4* and its isotypes *C4A* and *C4B* in cases and controls are shown in Table 2. Four copies of total *C4* and two copies of *C4A* and C4B were the most common findings both in patients and controls. The distribution of total *C4* and *C4A* differed significantly between cases and controls with an apparent shift to lower copy numbers in the patient group (total *C4*: *p* = 9.2 × 10^−3^; *p*_adjusted_ = 0.028; *C4A*: *p* = 2.1 × 10^−4^; *p*_adjusted_ = 6.3 × 10^−4^), whereas no significant effect was observed in *C4B* (*p* = 0.062, *p*_adjusted_ = 0.185). *C4B* was therefore not further investigated.

**Table 2:** Distribution of *C4* gene copy numbers in patients and controls

Total *C4*	Two copies	Three copies	Four copies	Five copies	Six copies
SLE (*n* = 160)	11 (6.9%)	59 (36.9%)	74 (46.3%)	15 (9.4%)	1 (0.6%)
Controls (*n* = 460)	9 (1.9%)	135 (29.4%)	254 (55.2%)	58 (12.6%)	4 (0.9%)
Fisher’s exact test:					*p* = 9.2 × 10^−3^
Bonferroni’s correction:					*p* = 0.028
C4A	*Zero copies*	*One copy*	*Two copies*	*Three copies*	*Four copies*
SLE (*n* = 163)	7 (4.3%)	54 (33.1%)	79 (48.5%)	22 (13.5%)	1 (0.6%)
Controls (*n* = 479)	4 (0.8%)	101 (21.1%)	260 (54.3%)	101 (21.1%)	13 (2.7%)
Fisher’s exact test:					*p* = 2.1 × 10^−4^
Bonferroni’s correction:					*p* = 6.3 × 10^−4^
C4B	*Zero copies*	*One copy*	*Two copies*	*Three copies*	*Four copies*
SLE (*n* = 164)	0 (0.0%)	28 (17.1%)	129 (78.7%)	7 (4.3%)	0 (0.0%)
Controls (*n* = 473)	11 (2.3%)	109 (23.0%)	335 (70.8%)	18 (3.8%)	0 (0.0%)
Overall testing (Fisher’s exact test):					*p* = 0.062
Bonferroni’s correction:					*p* = 0.185
	*Copies*	*Odds ratio*	*95% CI*	p *value*	*Bonferroni’s correction*
Total *C4*	2 vs. >2	3.699	1.504–9.101	6.7 × 10^−3^	0.034
	≥5 vs. <5	0.713	0.399–1.276	0.272	1.00
*C4A*	0 vs. >0	5.329	1.539–18.445	7.7 × 10^−3^	0.039
	≤1 vs. >1	2.130	1.451–3.128	1.8 × 10^−4^	8.8 × 10^−4^
	≥3 vs. <3	0.526	0.323–0.857	0.011	0.053

The frequencies of gene copy numbers for total *C4*, *C4A* and *C4B* are shown in patients and controls. Testing for independence was performed using Fisher’s exact test. *P* values for overall testing were adjusted for multiple testing with Bonferroni’s correction (*n* = 3 tests). *P* values for odds ratios were adjusted for multiple testing with Bonferroni’s correction (*n* = 5 tests).

SLE: systemic lupus erythematosus; CI: confidence interval.

Individuals carrying only two copies for total *C4* were at risk for SLE (OR = 3.699; 95% confidence interval (95% CI) = 1.504–9.101; *p* = 6.7 × 10^−3^, *p*_adjusted_ = 0.034). A protective effect for five and six copies could not be found in our data (OR = 0.713; 95% CI = 0.399–1.276; *p* = 0.272, *p*_adjusted_ = 1.00). Homozygous deficiency of *C4A* was identified as the strongest risk factor in our cohort (OR = 5.329; 95% CI = 1.539–18.445; *p* = 7.7 × 10^−3^, *p*_adjusted_ = 0.039), followed by the combined group including homozygous-deficient individuals and patients with one copy of *C4A*, i.e. heterozygous deficiency (OR = 2.13; 95% CI = 1.451–3.128; *p* = 1.8 × 10^−4^; *p*_adjusted_ = 8.8 × 10^−4^). No effect could be shown for more than three copies of *C4A* after adjusting for multiple testing (OR = 0.526; 95% CI = 0.323–0.857; *p* = 0.011; *p*_adjusted_ = 0.053).

### SNP analysis

Results from SNP analysis are shown in Table 3. All SNPs showed no deviation from Hardy-Weinberg equilibrium. The SNPs rs558702 (intronic C2 region) and rs2187668 (human leukocyte antigen (HLA)-DRB1*0301/HLA-DR3 tag SNP) were associated with SLE in the allelic and dominant model (OR = 2.290; 95% CI = 1.579–3.322; *p* = 9.63 × 10^−6^ and OR = 2.231; 95% CI = 1.548–3.216; *p* = 1.33 × 10^−5^, respectively), whereas rs3135391 (HLA-DRB1*1501/HLA-DR2 tag SNP) was not associated with SLE overall. The SNPs rs558702 (intronic C2 region) and rs2187668 (HLA-DRB1*0301/HLA-DR3 tag SNP) were in moderate to strong linkage disequilibrium (*D*’ = 0.87; *r*^2 ^= 0.7).

**Table 3:** SNP results: Minor allele frequencies and inheritance models

(a) Minor allele frequency									
	Patients		Controls	Minor allele			Location		
rs558702	21.43%		11.82%	A			Intronic *C2* region		
rs2187668	22.19%		13.13%	A			HLA-DRB1*0301 (DR3) tag SNP		
rs3135391	16.16%		13.84%	T			HLA-DRB1*1501 (DR2) tag SNP		
*(b) Inheritance models*									
	*Allelic model*			*Dominant model*			*Recessive model*		
*SNP*	*OR*	*95% CI*	p (χ^*2*^)	*OR*	*95% CI*	p (χ*^2^*)	*OR*	*95% CI*	p (χ*^2^*)
rs558702	2.035	1.474–2.809	1.18 × 10^−5^	2.290	1.579–3.322	9.63 × 10^−6^	2.587	0.779–8.588	0.108
rs2187668	1.886	1.378–2.582	6.21 × 10^−5^	2.231	1.548–3.216	1.33 × 10^−5^	1.36	0.413–4.475	0.611
rs3135391	1.2	0.851–1.691	0.298	1.309	0.886–1.933	0.176	0.777	0.256–2.356	0.654

Results from SNP analysis with minor allele frequencies and inheritance models are shown. *P* values were calculated with χ^2^ test.

SNP: single-nucleotide polymorphism; HLA: human leukocyte antigen; OR: odds ratio; CI: confidence interval.

When considering jointly *C4* or *C4A* copy number and SNPs as influence variables for SLE status, only SNP rs558702 (intronic C2 region) remained in the model and the copy numbers were no longer significant (data not shown).

### *C4* serum levels

**Figure 1:**
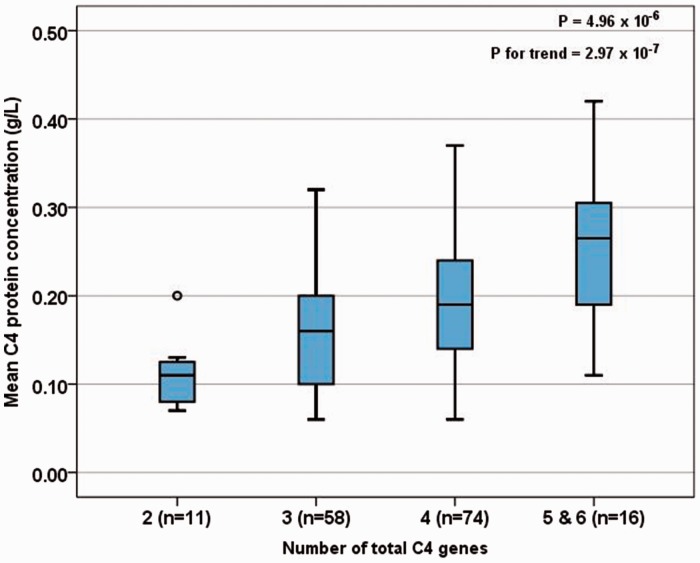
Association of *C4* gene copy numbers and C4 protein levels. The mean serum *C4* concentration measured during a 12-month period prior to genetic analysis and its relationship to *C4* gene copy numbers. *P* values from the Kruskal-Wallis test and Jonckheere-Terpstra test (*p* for trend). The length of the box shows the interquartile range. The band within the box indicates the median of the group. The ends of the whiskers identify the upper and the lower 1.5-fold interquartile range, respectively. The circle in group 2 denotes an outlier.

To determine the *C4* serum concentration, we calculated the mean of at least five serum samples obtained from every patient during the 12-month period prior to genetic analysis. The mean *C4* serum concentration was highly related to the *C4* gene copy numbers and increased from 0.110 g/l (two copies) over 0.157 g/l (three copies) and 0.189 g/l (four copies) to 0.256 g/l (five and six copies). The Kruskal-Wallis test revealed a significant association (*p* = 4.96 × 10^−6^) with a substantial trend (*p*_for trend_ = 2.97 × 10^−7^). Detailed results are shown in Figure 1.

### Therapeutic grading

Because of the highly unequal sizes of therapeutic groups (see Table 1), all patients from grade 1 to 5 were summarized and compared to the group of patients who had received cyclophosphamide treatment. Results are shown in Table 4.

**Table 4:** Frequencies of *C4* gene copy numbers in two groups of patients with different treatment

Total C4	Two copies	Three copies	Four copies	Five copies	Six copies
Patients with cyclophosphamide treatment (*n* = 52)	7 (13.5%)	17 (32.7%)	22 (42.3%)	5 (9.6%)	1 (1.9%)
Patients without cyclophosphamide treatment (*n* = 108)	4 (3.7%)	42 (38.9%)	52 (48.1%)	10 (9.3%)	0 (0.0%)
	Overall testing (Fisher’s exact test):				*p* = 0.108
	Bonferroni’s correction:				*p* = 0.216
*C4A*	*Zero copies*	*One copy*	*Two copies*	*Three copies*	*Four copies*
Patients with cyclophosphamide treatment (*n* = 52)	5 (9.6%)	17 (32.7%)	23 (44.2%)	7 (13.5%)	0 (0.0%)
Patients without cyclophosphamide treatment (*n* = 111)	2 (1.8%)	37 (33.3%)	56 (50.5%)	15 (13.5%)	1 (0.9%)
Overall testing (Fisher’s exact test):					*p* = 0.226
Bonferroni’s correction:					*p* = 0.452
	*Copies*	*Odds ratio*	*95% CI*	p *value*	*Bonferroni’s correction*
Total *C4*	2 vs. >2	4.044	1.128–14.507	0.040	0.120
*C4A*	0 vs. >0 ≤1 vs. >1	5.789 1.345	1.086–30.957 0.690–2.657	0.034 0.391	0.102 1.00

The distribution of *C4* gene copy numbers in patients who had received cyclophosphamide treatment and all other patients who received less aggressive treatment are shown. *P* values for overall testing were calculated with Fisher’s exact test and adjusted for multiple testing with Bonferroni’s correction (*n* = 2 tests). *P* values for odds ratios were calculated with Fisher’s exact test and adjusted for multiple testing with Bonferroni’s correction (*n* = 3 tests).

CI: confidence interval.

Similar to the association in general, four copies of total *C4* and two copies of *C4A* were the most frequently detected copy numbers in both groups. The group receiving cyclophosphamide treatment included slightly more patients with only two copies of total *C4* and 0 copies of *C4A*, but in overall testing, there was no significant difference in the general distribution of gene copy numbers between the patients receiving cyclophosphamide and the patients with less aggressive therapy (total *C4*: *p* = 0.108; *p*_adjusted_ = 0.216; *C4A*: *p* = 0.226; *p*_adjusted_ = 0.452). Comparing the subgroups of patients, individuals carrying only two copies for total *C4* had more often been exposed to cyclophosphamide therapy (OR = 4.044; 95% CI = 1.128–14.507; *p* = 0.040, *p*_adjusted_ = 0.120) and individuals with homozygous deficiency of *C4A* had an even higher exposure to cyclophosphamide treatment (OR = 5.798; 95% CI = 1.086–30.957; *p* = 0.034, *p*_adjusted_ = 0.102) even though the results remained no longer significant after adjustment for multiple testing. Summarizing the patients carrying homozygous and heterozygous deficiency of *C4A*, no significant exposure to cyclophosphamide therapy could be detected (OR = 1.345; 95% CI = 0.690–2.657; *p* = 0.391; *p*_adjusted_ = 1.00).

### Age at diagnosis

Age at diagnosis was significantly associated with total *C4* and *C4A* gene copy numbers even after adjustment for multiple testing (total *C4*: *p* = 7.2 × 10^−3^, *p*_adjusted_ = 0.014; *C4A*: *p* = 2.8 × 10^−3^, *p*_adjusted_ = 5.6 × 10^−3^) without a trend. Detailed results are shown in Table 5.

**Table 5:** Age at first diagnosis and *C4* gene copy numbers

Age at first diagnosis	Gene copy numbers of total *C4*			
	Two (*n* = 11)	Three (*n* = 58)	Four (*n* = 74)	Five and six (*n* = 16)
Median	25	39	31	35
Mean	29.18	40.81	33.07	39.44
Range	19–45	15–71	14–60	16–69
25% quartile	20	29.25	23.75	25.25
75% quartile	41	53	42.25	52.50
	Kruskal-Wallis test: Bonferroni’s correction:			*p* = 7.2 × 10^−3^ *p* = 0.014
	*Gene copy numbers of* C4A			
*Age at first diagnosis*	*Zero* *(*n = *7)*	*One* *(*n = *53)*	*Two (*n = *79)*	*Three and four (*n = *23)*
Median	24	41	31	41
Mean	25.43	41.21	33.52	38.26
Range	19–42	17–71	14–69	16–67
25% quartile	20	28.50	24	25
75% quartile	28	53	41	48
Kruskal-Wallis test:				*p* = 2.8 × 10^−3^
Bonferroni’s correction:				*p* = 5.6 × 10^−3^
	*Copies*	*Median age at diagnosis*	p *value*	*Bonferroni’s correction*
Total *C4*	2 vs. >2	25 vs. 34	0.069	0.207
*C4A*	0 vs. >0 ≤1 vs. >1	24 vs. 34 37 vs. 32.5	0.019 0.073	0.057 0.219

The age at first diagnosis of SLE in relation to the *C4* gene copy numbers are shown. *P* values were calculated using Kruskal-Wallis test and adjusted for multiple testing with Bonferroni’s correction (*n* = 2 tests). *P* values for grouped copy numbers were calculated with Mann-Whitney-*U* test and adjusted for multiple testing with Bonferroni’s correction (*n* = 3 tests).

SLE: systemic lupus erythematosus.

Patients with homozygous deficiency of *C4A* were a median of 10 years younger in age at diagnosis compared to patients without homozygous *C4A* null alleles (median 24 vs. 34 years; *p* = 0.019, *p*_adjusted_ = 0.057). Patients with two copies of total *C4* showed a similar trend that was, however, not clear enough to reach statistical significance (median 25 vs. 34 years; *p* = 0.069, *p*_adjusted_ = 0.207). The group containing patients with 0 and one copy of *C4A* did not significantly differ from all other patients (median 37 vs. 32.5 years; *p* = 0.073; *p*_adjusted_ = 0.219).

## Discussion

We investigated the complement *C4* gene locus to evaluate the impact of the individual genetic variations on the clinical course of SLE. Our findings confirm the association of low *C4* gene copy numbers and of homozygous *C4A* deficiency with SLE. Although complete deficiency of C4A was detected in a minority of both groups, its impressive impact on the risk of SLE emphasizes the important role of *C4* in the susceptibility of SLE. The more than fivefold increased risk of SLE in *C4A*-deficient individuals is well in line with similar findings in other SLE cohorts.^3,4,10^

As a new finding, low copy numbers of total *C4* and homozygous deficiency of *C4A* were associated with a distinctly higher rate of exposure to cyclophosphamide therapy (although not significant after adjustment for multiple testing), implicating that these genetic states may predispose to a more severe and aggressive course of the disease.

As an additional aspect, the role of complement *C4* is underscored by our finding of an association between complete *C4A* deficiency and an earlier onset of SLE. To our knowledge, this is the first genetic study demonstrating a median 10 years’ difference in the age of first diagnosis between individuals completely lacking *C4A* and the remaining patients.

Taken together, our results support hypotheses suggesting a strong immunological effect of complement *C4* both on the general risk of systemic lupus as well as on the clinical course of the disease.

Moreover, the role of *C4* copy numbers is substantiated by our finding of a clear and highly significant relationship between complement *C4* copy numbers and *C4* serum levels. This correlation has repeatedly been described in healthy individuals,^11–13^ but has rarely been reported in lupus patients.^14^ In the study presented here, the *C4* protein levels represent a mean of an average of five samples taken over a period of one year. Therefore, the chance of misinterpreting individual disease flares associated with complement consumption is low.

The importance of complement *C4* in the immunobiology of systemic lupus has been widely studied.^15,16^ As a substantial component of the classical complement pathway, *C4* is involved in the clearance of immune complexes as well as in the clearance of apoptotic cell bodies.^17,18^ This has been repeatedly demonstrated in complement-deficient individuals, as well as by in vitro experiments, showing that *C4* promotes the opsonization and the clearance of immune complexes in concert with complement *C3* in a dose-dependent manner.^19^ *C4A* appears to play a more important role than *C4B* among the *C4* isotypes, based on its acidic chemical properties and higher affinity to immunoglobulins.^20,21^ Defects in those processes have been observed in patients suffering from SLE as well as in other disorders classified as immune complex-mediated diseases.^22,23^ In addition, a state of *C4* deficiency has been shown to affect the regulation and suppression of B-cell tolerance, leading to an increased survival of autoreactive B-cells.^24,25^

However, recent findings suggest that low copy numbers of *C4* are not an independent risk factor for SLE, but that they are related to other SLE risk loci such as HLA-DRB1*0301 (DR3 isotype) or HLA-DRB1*1501 (DR2 isotype) located inside the MHC.^6^ Indeed, the association of SLE and HLA-DRB1*0301 was observed in our patients and this finding is consistent with common genetic features of other Northern European lupus samples.^26,27^ In contrast, HLA-DRB1*1501 did not increase the risk for SLE in our cohort. These findings are in accord with studies indicating that the association with HLA-DRB1*1501/HLA-DR2 is either less strong^28,29^ or even not significantly increased^30^ in Caucasian or Hispanic cohorts, whereas the strongest evidence of the role of HLA-DR2 in SLE appears to be found in patients of Asian origin.^31^

In a logistic regression considering the joint effect of *C4* and *C4A* copy number variation and of the HLA genotypes and rs558702 (intronic region of the complement *C2* gene) as influence variables on SLE, neither *C4* status nor HLA genotypes remained significant but only rs558702 did. However, note the substantial linkage disequilibrium between rs558702 and HLA-DRB1*0301 (*r*^2 ^= 0.7).

Thus, our findings confirm the linkage between the gene loci of *C4* and of HLA-DRB1*0301, an association which primarily appears to reflect a highly conserved ancestral haplotype in European lupus patients.^32^ Despite the reproducibility of these findings, the relative weight of either component appears to be less clear. Indeed, there are several findings of genetic or functional *C4A* null alleles in patients with SLE not carrying the HLA-DRB1*0301 genotype.^2,33^ The predominant HLA alleles appear to differ among global patient groups with various ethnic backgrounds, especially in nonwhite populations and in patients of non-European ancestry.^30,34^ In contrast, the C4A null allele appears not to be restricted to certain ethnicities in general.^2,35^

As an additional aspect, the implication of HLA-DR3 for the susceptibility to SLE is not substantiated by a known or proven pathophysiological mechanism supporting the role of this gene locus. Whereas the HLA genes identified in rheumatoid arthritis or celiac disease have been shown to be involved in direct or indirect molecular interactions with the presumable disease-associated antigen,^36,37^ similar findings have not been described for HLA-DR3 and the known autoantigens of SLE.

The association of rs558702 and SLE has recently been reported^38^ but, because of its localization inside an intronic region of the complement C2 gene, its pathogenetic role is completely unclear. The demonstrated linkage disequilibrium in our findings suggests that rs558702 might be a concomitant feature of HLA-DRB1*0301 or it might be an indicator SNP of another risk locus inside the MHC that has not been detected yet.

In conclusion, the strong association of SLE in individuals with low copy numbers of *C4* and in particular in patients with complete deficiency of *C4A* was confirmed by our data. In this lupus cohort, patients carrying the homozygous *C4A* null allele were exposed to an earlier onset of the disease and to the requirement of cyclophosphamide treatment, suggesting a more aggressive course of the disease. However, some of these associations were not significant after correction for multiple testing, probably arising from the sample size of only 169 patients in this single-center study. Replication studies with larger sample sizes are recommended to verify these novel results. Furthermore, it is difficult to estimate the severity of SLE. Given the heterogeneous patient group with several individuals suffering from SLE for more than 30 years, the commonly used assessment tools like the Systemic Lupus Erythematosus Disease Activity Index (SLEDAI) or Systemic Lupus International Collaborating Clinics Damage Index (SLICC) were not available for all patients. In the absence of other parameters, we used the most intensive treatment procedures ever applied as an approach to assess the severity of the disease in our study because the choice of the drug is determined largely by the severity of the disease and the major organ involvement.^39^ With respect to this approach, the highest grade of severity was attributed to the patients with a disease course requiring cyclophosphamide treatment since cyclophosphamide was regarded as the most intensive treatment option for severe lupus for two decades before the introduction of mycophenolate and is still of high importance in the appropriate indications.^40,41^ Further studies with commonly used damage indices are required to prove the associations shown here.

Summarizing these findings, we suggest that the biologic effects of low C4 copy numbers and homozygous deficiency of C4A may influence the clinical course of SLE and should be considered in the management of patients with SLE.
